# Lexical adaptation of link grammar to the biomedical sublanguage: a comparative evaluation of three approaches

**DOI:** 10.1186/1471-2105-7-S3-S2

**Published:** 2006-11-24

**Authors:** Sampo Pyysalo, Tapio Salakoski, Sophie Aubin, Adeline Nazarenko

**Affiliations:** 1 Turku Centre for Computer Science (TUCS) and University of Turku, Lemminkäisenkatu 14 A, 20520 Turku, Finland; 2 LIPN, Université Paris 13 & CNRS UMR 7030, 99, av. J.-B. Clément, F-93430 Villetaneuse, France

## Abstract

**Background:**

We study the adaptation of Link Grammar Parser to the biomedical sublanguage with a focus on domain terms not found in a general parser lexicon. Using two biomedical corpora, we implement and evaluate three approaches to addressing unknown words: automatic lexicon expansion, the use of morphological clues, and disambiguation using a part-of-speech tagger. We evaluate each approach separately for its effect on parsing performance and consider combinations of these approaches.

**Results:**

In addition to a 45% increase in parsing efficiency, we find that the best approach, incorporating information from a domain part-of-speech tagger, offers a statistically significant 10% relative decrease in error.

**Conclusion:**

When available, a high-quality domain part-of-speech tagger is the best solution to unknown word issues in the domain adaptation of a general parser. In the absence of such a resource, surface clues can provide remarkably good coverage and performance when tuned to the domain. The adapted parser is available under an open-source license.

## Background

In applying general parsers to specific domains, adaptation is often necessary to achieve high parsing performance (see e.g. [1]). Sublanguage is defined by Grishman [2] as a specialized form of a natural language that is used within a particular domain or subject matter. It is characterized by specialized vocabulary, semantic relationships, and in many cases syntax.

In this paper, we study lexical adaptation, that is, adaptation addressing the specialized vocabulary. This is an important part of the process of customizing a general parser to a sublanguage. Among other issues, the unknown word rate increases dramatically when moving from general language to increasingly technical domains such as that of biomedicine [3]. This can lead to increased ambiguity, reduced parsing performance, and errors in establishing the correct relationships between words for semantic mining [4].

Until recently, most Information Extraction (IE) systems for mining semantic relationships from texts of technical sublanguages avoided full syntactic parsing. The quality of parsing has a well-established effect on the performance of IE systems, and the accuracy of general parsers in technical domains is comparatively low. Additionally, many domain-specific parsers lack portability to a new domain. Finally, the time required for full parsing is also a problem for IE systems. However, the biomedical IE community now faces limitations in pattern-matching [5] and shallow parsing [6] methods that are inefficient in the processing of long distance dependencies and complex sentences. Advances in parsing techniques have further created an increased interest in the adaptation of full parsers, and there have recently been several applications of full parsers in the biomedical domain [3,7-9].

Here, we consider the lexical adaptation of a full parser, the Link Grammar Parser (LGP) of Sleator and Temperley [10,11]. The choice of parser addresses the recent interest in LGP in the biomedical IE community [12-15]. Our evaluation is performed using two corpora of sentences from Medline abstracts with a focus on protein-protein interactions, the identification of which is the key aim of many biomedical IE systems.

Recently, two approaches addressing unknown words in applying LGP to the biomedical domain have been proposed. Szolovits [13] introduced a method for heuristically mapping terminology between lexicons and applied this mapping to augment the LGP dictionary with terms from the UMLS Specialist Lexicon [16]. Based on an analysis of a domain corpus, two of the authors have proposed an extension of the morpho-guessing system of LGP for disambiguating domain terms based on their suffixes [17]. The effect of the proposed extensions on parsing performance against an annotated reference corpus was not evaluated in these two studies. Here we analyze the effect of these lexical extensions using an annotated biomedical corpus. We further propose, implement and evaluate in detail a third approach to resolving unknown words in LGP using information from a part-of-speech (POS) tagger.

The evaluated lexical adaptation approaches address unknown single-word units: related adaptation issues such as multi-word expressions, grammar adaptation, text preprocessing, handling of complex terms, improved parse ranking and named entity recognition fall outside the scope of this study.

### Link grammar parsing

The lexical adaptation approaches we evaluate require only a light linguistic analysis of domain language, facilitating their application to domain adaptation. Similarly, as Link Grammar is rule-based and its parser makes no use of statistical methods, LGP is a good candidate for adaptation to new domains where annotated corpus data is rarely available.

**Figure 1:**
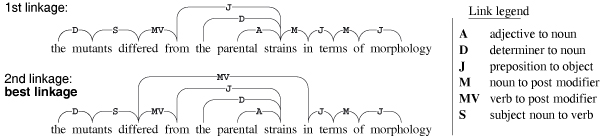
Example Link Grammar Parser linkages.

The Link Grammar formalism is closely related to dependency formalisms. It is based on the notion of typed *links *connecting words. The result of parsing is one or more ordered parses, termed *linkages*. A linkage consists of a set of links connecting the words of a sentence so that links do not cross, no two links connect the same two words, and the types of the links satisfy the *linking requirements *given to each word in the lexicon. For example, the linking requirements for a singular countable noun specify that the word must form a D link to the left to connect to a determiner. Two linkages for an example sentence are shown in Figure 1.

LGP has three different methods applied in a cascade to handle vocabulary: dictionary lookup, morpho-guessing and unknown word guessing. The LGP dictionary enumerates all words, including inflected forms, and grammar rules are encoded through the linking requirements associated with the words. Some unknown words are assigned linking requirements based on their morphological features, such as the suffix -*ly *for adverbs. This system is termed *morpho-guessing *(MG). Finally, words that are neither found in the parser dictionary nor recognized by its morpho-guessing rules are assigned all possible combinations of the generic verb, noun and adjective linking requirements. This general approach is, in principle, always capable of generating the correct combination of linking requirements for unknown words. However, with an increasing number of unknown words in a sentence, the approach leads to a combinatorial explosion in the number of possible linkages and a rapid increase in parsing time and decrease in parsing performance. The parser is also time-limited: when a sentence cannot be parsed within a user-specified time limit, LGP attempts parses using more efficient, but restricted settings, leading to reduced parse quality. When parsing sublanguages that contain many words that are not in the lexicon, it is therefore beneficial to attempt to resolve unknown words to reduce ambiguity in parsing.

## Methods

We evaluate three approaches to lexical adaptation: lexicon extension, morphological clues, and POS tagging. The approaches primarily involve open-class words and use linking requirements from the original LGP. Closed-class words, such as prepositions, are considered domain-independent and expected to appear in the original lexicon, and we have chosen not to perform any modification of the existing linking requirements (grammar adaptation) in this study.

### Extension of the lexicon

The extension of the lexicon with external domain-specific knowledge is the most frequent approach to adaptation, provided that the resources are available for the domain. This can be done either manually or with automatic mapping methods.

Here, we evaluate the heuristic lexicon mapping proposed by Szolovits [13]. This mapping can be used to automatically add domain-specific terminology from an external specialized lexicon to the lexicon of a parser. Words are mapped from a source lexicon (e.g. the domain lexicon) to a target lexicon (e.g. the parser lexicon) based on their lexical descriptions. As these descriptions typically differ between lexicons, they cannot be transferred directly from one lexicon to another. Instead, the mapping operates with sets of words that have the exact same lexical description in their respective lexicons.

To assign a lexical description to a word *w *not in the target lexicon, the mapping finds words that have the exact same lexical description as *w *in the source lexicon, and that further have a description in the target lexicon. Overlap in sets having the same descriptions is then used to select one of these target lexicon descriptions to assign to *w*.

Szolovits applied the introduced mapping to extend the lexicon of LGP with terms from the UMLS Specialist Lexicon and observed that the mapping heuristic chose poor definitions for some smaller sets, for which the definitions were manually modified. The created dictionary extension contains 121,120 words that do not appear in the original LGP dictionary. The effect of adopting this extension has also been considered by Pyysalo et al. [4].

We chose to evaluate the version of the dictionary extension that does not include multi-word terms [18] for the following reasons. First, Szolovits observed that many of the phrases included in the extension "bear no specific lexical information in Specialist that is not obvious from their component words", suggesting that it is sufficient to include the component words in the parser lexicon separately. Additionally, multi-word phrases entered into the LGP lexicon are parsed using the LGP idiom system, which does not assign internal structure to the phrases. Thus, if the phrases were included, automatic comparison against a reference corpus containing phrase-internal structure would find missing links for the terms.

### Morphological clues

Morphological clues can be exploited by LGP to predict the morpho-syntactic classes (and hence syntactic behaviour) of unknown words. Specific domains are an interesting application for this type of adaptation because a great part of technical lexicons presents regular morphological features, which, according to Mikheev [19], obey morphological regularities of the general language. We observe that this assumption holds only partially because of the presence of foreign words in specialized texts and argue that a minimal morphological study of the corpus is necessary. Such studies have been performed, on the biomedical domain by Spyns [20] and Aubin et al. [17].

While many POS taggers employ morphological features to tag unknown words, domain extension of a rule-based approach such as the LGP morpho-guessing system can be preferable in lexical adaptation to domains where resources such as tagged corpora are not available for training taggers. Further, the MG extension allows assigning specific rules at a finer granularity than POS tags.

We have implemented and evaluated the extension of the LGP morpho-guessing rules proposed by Aubin et al. [17]. This extension of 23 new suffixes for the biomedical domain is presented in Table 1. Aubin et al. further identified a small number of exceptions to these rules (*wherein*, *kcal/mol*, *ultrafine*, etc.), which were manually added to the dictionary.

### POS tagging

Finally, we propose to provide the parser with an input sentence enriched with POS tags. In order to retain the decision-making power of the parser and to avoid inconsistencies between tagged words and their entry in the parser lexicon (see Grover et al. [21]), we restrict the use of POS tags to unknown words only.

We modified LGP so that POS information can be passed to the parser by appending POS tags to input words (e.g. *actin/NN*). We further modified the parser so that when an unknown word is given a POS tag, the parser assigns linking requirements to the words based on a given mapping from POS tags to LGP dictionary entries. We defined such a mapping, presented in Table 2, for Penn tagset POS categories corresponding to content words. FW (foreign words) and SYM (symbols) tags were not mapped due to their syntactic heterogeneity. Existing LGP rules were used to define the behaviour of POS-mapped words, and the most generic applicable rule was chosen in each case. For instance, words tagged *NN* map to the rule for nouns that can be either mass or countable, so that there is no constraint on determiners.

To evaluate the effect of using both a tagger for general language and a tagger for domain language, the experiments were made using two taggers: the Brill tagger [22] trained on the Wall Street Journal (general language) and the GENIA Tagger (version 2.0.1) [23,24] trained on a combination of the Wall Street Journal and the biomedical corpora GENIA [25] and PennBioIE [26]. Note that in comparing taggers trained on different resources, we observe both effects relating to the training corpus and effects relating to the performance of the tagger.

A detailed evaluation and error analysis of GENIA Tagger is given in [24], finding 98% accuracy on two biomedical corpora. As part of the present study, a linguist evaluated the subset of words in the interaction corpus that are handled by the POS-mapping method and show tagging divergence between the two taggers (350 words). On the basis of this comparison and the reported performance of GENIA Tagger, we estimate that for the subset of words that are handled by the POS-mapping method, the tagging accuracy is 81% for the Brill tagger and 97% for GENIA Tagger.

### Evaluation protocol

Two corpora are used for the present evaluation: "interaction" and "transcript", both built in the context of IE from biomedical texts. Both corpora were tokenized and cleared of bibliographic references in a preprocessing step.

Interaction contains 542 sentences (16,874 tokens) annotated for dependencies using the Link Grammar annotation scheme. 600 sentences were initially selected randomly from Pubmed with the condition that they contain at least two proteins for which a known interaction was entered into the DIP database [27]. 58 sentences consisting only of a nominal phrase were then excluded as the LGP grammar is only designed to analyse full clauses – this limitation could be overcome by modification of the grammar, but here we decided to avoid grammar adaptation and evaluate LGP with respect to its intended coverage. Each sentence was separately annotated by two annotators, and differences were resolved by discussion. Links to punctuation were excluded, and link types were not annotated. A total of 14,242 links were annotated in these sentences.

The transcript corpus is made of 16,989 sentences (438,390 tokens) consisting of the result for the query "*Bacillus subtilis transcription*" on Pubmed. It was not annotated.

Both corpora are used to characterize the vocabulary coverage by the different methods applied in LGP. The annotated interaction corpus is also used as the reference corpus for the evaluation of parsing performance. Aubin et al. [17] used the transcript corpus in defining the MG extension rules. By contrast, the interaction corpus, used here to evaluate performance, is a blind test set with respect to all evaluated extensions.

We first evaluate *vocabulary coverage *in the original and extended versions of LGP. We present the contribution of each method (dictionary, morpho-guessing, POS-mapping and unknown words) implemented in LGP to handle vocabulary. Results are given separately for types (i.e. distinct forms) and tokens (i.e. occurrences) in the corpus.

We assess the *ambiguity of the parsing *process with two criteria: parsing time and linkage numbers. Parsing time is immediately relevant to applications of the parser to systems where large corpora must be parsed. Linkage numbers are a more direct measure of the ambiguity of parsing a sentence. For each sentence, the parser enumerates the total number of linkages allowed by the grammar. By taking the ratio of the number of linkages allowed by two versions of the parser, we can estimate the relative increase or decrease in ambiguity. We report the per-sentence averages of both parsing time and linkage number ratios.

To determine the *parsing performance *of the extensions of LGP, we used each of the extensions to parse the interaction corpus sentences and compared the produced linkages against the reference corpus. For each sentence, we determine the recall, i.e. the fraction of links in the reference corpus that were present in parses returned by LGP. Note that for connected, acyclic dependency graphs, precision equals recall: for each missing link, there is exactly one extra link. While there are some exceptions to connectedness and acyclicity in both LGP linkages and the annotation, we believe recall can be used as a fair estimate of overall performance. For each sentence, we measure recall for both the *first linkage *as ordered by the LGP heuristics and, to separate the effect of the heuristics from parser performance, also the *best linkage*, that is, the linkage that is most correct with respect to the annotated corpus (see Figure 1). As parse reranking methods can be used to improve over the LGP heuristics in parse ordering [28,29], it is also important that best linkage performance is not decreased in adaptation. We further separately evaluate overall performance and performance for the subset of sentences where no timeouts occurred in parsing.

Experiments were performed on a 2.8 GHz Intel Xeon with parameter values timeout = 60 sec, limit = 1000, islands-ok = true. Default values were used for other parameters. The statistical significance of differences between the original parser and each of the modifications is assessed using the Wilcoxon signed-ranks test [30] for overall first linkage performance, using the Bonferroni correction for multiple comparisons, following the recent recommendation of Demšar [31].

## Results

In this section we present the evaluation results for the original LGP (Orig), LGP with the UMLS Specialist dictionary extension (UMLS), LGP with the morpho-guessing extension (xMG) and LGP with the POS extension, evaluated with the two taggers, Brill and GENIA Tagger (GT).

### Vocabulary coverage

**Figure 2:**
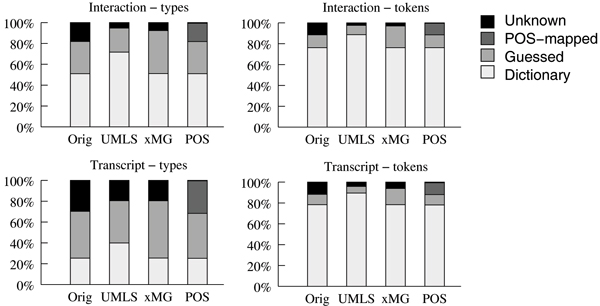
Vocabulary handling in the interaction and transcript corpora.

Figure 2 shows the proportion of vocabulary covered by each method on the interaction and transcript corpora. Coverage for the POS adaptation is shown only for GENIA Tagger as the coverage of the Brill tagger was essentially identical.

The comparison of the results on types and tokens shows that the dictionary has a good recognition rate on frequent types for both the original and the UMLS versions. By contrast, the MG and POS-map methods contribute to the recognition of a great number of types (particularly in transcript) but few tokens. In addition, the discrepancy in types between the two corpora for the dictionary method in all versions reflects the increasing presence of low-frequency non-canonical words with the growing size of the corpus. Interestingly, we find that the reduction in unknown words due to the UMLS and xMG extensions is roughly similar, despite the former containing over 100,000 new words and the latter only 23 new rules. The POS extension, as expected, reduces the part of unknown words to almost null.

The nature of the words that remain unknown varies depending on the extension. Quite surprisingly, UMLS Specialist lacks a great number of species names (numerous in transcript) and frequent gene or protein names (e.g. *lacZ*, 78 occurrences in transcript). In addition, the version of the dictionary extension used here contains no multi-word terms, which prevents the detection of words like *vitro *and *vivo *used in the frequent terms *in vitro *and *in vivo*. The evaluated xMG extension cannot handle gene/protein names either, and also misses frequent technical terms that have no specific morphological features, such as *sigma*, *mutant *and *plasmid*.

To assess lexicon coverage, we measured the *contribution *(proportion of types of the resource found in the corpus) and the *recognition *(proportion of types of the corpus found in the resource) of the UMLS Specialist dictionary extension. We find that while the contribution of the UMLS extension is very low, with 0.54% on interaction and 2.3% on transcript, the recognition of the dictionary method is augmented significantly by the UMLS extension (51% to 71% for interaction and 25% to 40% for transcript). Nevertheless, as the size of the dictionary does not significantly penalize the parsing time with LGP, even a generic resource that contributes relatively little can be beneficial.

### Ambiguity

The results of measuring the effect of the various extensions on ambiguity are given in Table 3.

The reduction in the number of unknown words for the UMLS and xMG extensions is coupled with a roughly 30% reduction in both parsing time and linkage numbers. Although the POS extension essentially eliminates unknown words, it only gives a decrease in parsing time and linkage numbers that roughly mirrors the effect of the UMLS and xMG extensions.

None of the extensions achieves more than 35% reduction in linkage numbers or more than 45% reduction in parsing time. This may reflect structural ambiguity in the language and suggest a limit on how much ambiguity can be controlled through these lexical adaptation approaches.

### Performance

The evaluation results are presented in Table 4. We find that in addition to increased efficiency, all of the extensions offer an increase in overall parsing performance compared to the original LGP for both the first and best linkages. Remarkably, this increase occurs even with the Brill tagger, which was trained on general English. In overall performance, the UMLS extension and the POS extension with the Brill tagger are roughly equal. The xMG extension outperforms both, and the POS extension with GENIA Tagger has the best performance of all considered extensions.

The positive effect of the extensions on parsing performance is linked to the reduced number of timeouts that occurred when parsing. Effects not related to time limitations can be studied on sentences where no timeouts occurred (NT). Here the effects of the extensions diverge: for the first linkage, performance with the UMLS extension and the POS extension with the Brill tagger essentially matches that of the unmodified LGP, while performance with xMG and GENIA Tagger remains better. For the best linkage, we observe a negative effect from the UMLS extension, indicating that for some words the unknown word handling mechanism of LGP finds correct links that are not allowed by the linking requirements given to those words in the extended dictionary. This suggest that some errors have occurred in the automatic mapping process. An example of one such error is in the mapping of abbreviations (e.g. *MHC*) to countable nouns, leading to failures to parse in the absence of determiners. We similarly observe the expected decrease in performance for the Brill tagger for the best linkage, reflecting tagging errors.

Even for the best linkage in sentences where no timeouts occurred, the performance with the xMG extension and the POS extension with GENIA Tagger is better than that of the original LGP. These extensions can thus assign more appropriate linking requirements for some words than the unknown word system of LGP. This indicates high tagging accuracy for GENIA Tagger as well as an appropriate choice of linking requirements for both extensions, and suggests some limitation in the unknown word system of LGP.

Despite significant improvements in parsing performance, the best performance achieved by any LGP extension is 88%. This may again suggest a limit on what performance can be achieved through the lexical adaptation approaches.

### Combinations of the extensions

The UMLS, xMG and POS tagging extensions are to some extent complementary as their coverage of the corpus vocabulary does not completely overlap. The UMLS extension provides the most frequent domain-specific lexical items while the xMG extension has the advantage of being able to handle non-canonical (e.g. *mutation/deletion*, *DNA-regions*) and rare words and misspellings. The POS extension can benefit from the context-sensitiveness of the tagger to disambiguate words.

We evaluated all possible combinations of the three extensions. In these experiments we only used GENIA Tagger for the POS extension. The results are given in Tables 5 and 6.

On ambiguity, we observe small advantages for many of the combinations, but rarely more than a 10% reduction for either metric compared to the simple extensions. The effect of the combinations on overall performance is mixed. While all combinations outperform the original LGP, combinations involving the UMLS extension appear to perform worse than those that do not, while combinations involving the xMG and POS extensions perform better. For sentences where no timeouts occurred the effect is simple: for the best linkage, all combinations involving the UMLS extension perform worse than the original LGP; only the combination of the xMG and POS extensions is better.

The performance of the best combination approach essentially matches that of the POS extension with GENIA Tagger alone, suggesting that no further benefit can be derived from combinations when an accurate domain tagger is available.

## Conclusion

We have studied three lexical adaptation approaches addressing biomedical domain vocabulary not found in the lexicon of the Link Grammar Parser: automatic lexicon expansion, surface clue based morpho-guessing, and the use of a POS tagger. We found that in a time-limited setting, any approach resolving unknown words can improve efficiency and overall performance. In more detailed evaluation, we found that the automatic dictionary extension and the use of a general English POS tagger can reduce performance, while the morpho-guessing approach and the use of a domain-specific POS tagger had only positive effects. We found no further benefit from combinations of the three approaches.

Generally, our results suggest that when available, a high-quality domain POS tagger is the best solution to unknown word issues in the domain adaptation of a general parser, here providing an overall 10% relative reduction in error combined with a 45% decrease in parsing time. We note that a comparable 14% reduction was also achieved by Lease and Charniak through POS adaptation for a statistical constituency parser [3]. This further enforces our conclusion on the value of accurate POS tags in support of the parsing process.

In the absence of a domain POS tagger, the use of a general POS tagger is a poor substitute, and can lead to decreased performance. The use of heuristic methods for lexicon expansion carries the risk of mapping errors and should be accompanied by an evaluation of the effect on parsing performance. Conversely, surface clues can provide remarkably good coverage and performance when tuned to the domain, here using as few as 23 new rules.

Our implementation of the adaptations to LGP combines the morpho-guessing extension with the capability of using information from a POS tagger. Thus, the adapted parser is faster and more accurate than the unmodified LGP in parsing biomedical texts both when used as such and when used together with a domain POS tagger. Further, both extensions are implemented so that defining other morpho-guessing rules and POS-mappings is straightforward, facilitating adaptation of the modified parser to other domains. The adapted LGP is available under an open-source licence at [32].

While we found that the considered approaches can significantly improve efficiency and parsing performance, our results also indicate some limitations for lexical adaptation. As future work, complementary approaches addressing multi-word expressions, grammar adaptation, text preprocessing, handling of complex terms, improved parse ranking and named entity recognition can be considered to further improve the applicability of LGP to the biomedical domain.

## Figures and Tables

**Table 1:** Biomedical suffixes involved in the extension of the morpho-guessing rules

**suffix**	**POS**	**examples**	**suffix**	**POS**	**examples**
*-ase*	noun	synthase, kinase	*-in*	noun	actin, kanamycin
*-ity*	noun	chronicity, hypochromicity	*-ion*	noun	septation, reguion
*-on*	noun	replicon, intron	*-ol*	noun	glycosylphosphatidylinositol
*-ose*	noun	isomaltotetraose, isomaltotriose	*-or*	noun	cofactor, repressor/activator
*-yl*	noun	hydroxyethyl, hydroxymethyl	*-ine*	noun	5-(hydroxymethyl)-2'-deoxyuridine
*-ide*	noun	iodide, oligodeoxynucleotide	*-i*	noun	casei, lactococci, termini
*-ic*	adjective	glycolytic, ribonucleic, uronic	*-al*	adjective	ribosomal, ribsosomal
*-ive*	adjective	nonpermissive, thermosensitive	*-ar*	adjective	intermolecular, intramolecular
*-ble*	adjective	inducible, metastable	*-ous*	adjective	exogenous, heterologous
*-ae*	latin adj.	influenzae, tarentolae	*-us*	latin adj.	pentosaceus, luteus, carnosus
*-um*	latin adj.	japonicum, tabacum, xylinum	*-is*	latin adj.	brevis, israelensis
*-fold*	adj./adv.	10-fold, 4.5-fold, five-fold			

**Table 2:** POS tag mapping to LGP rules

**Tag**	**Description of tag**	**LGP rule**	**Description of rule class**
NN	common noun, sing.	words.n.4	singular nouns that can be mass or countable
NNS	common noun, pl.	words.n.2.s	plural nouns ending in *"-s"*
NNP	proper noun, sing.	CAPITALIZED-WORDS	generic category for words with a capitalized first character
NNPS	proper noun, pl.	PL-CAPITALIZED-WORDS	capitalized words ending in *"-s"*
JJ	adjective, base	UNKNOWN-WORD.a	MG rule for adjectives
JJR	adjective, comparative	words.adj.2	comparative adjectives
JJS	adjective, superlative	words.adj.3	superlative adjectives
VB	verb, base	words.v.6.1	optionally transitive verbs (base form)
VBD	verb, past tense	words.v.6.3	optionally transitive verbs (*"-ed" *form)
VBZ	verb, present 3rd pers.	words.v.2.2	optionally transitive verbs (*"-s" *form)
VBP	verb, present non-3rd	words.v.6.1	optionally transitive verbs (base form)
VBG	verb, gerund	ING-WORDS	MG rule for words ending with *"-ing" *(noun or verb)
VBN	verb, past participle	ED-WORDS	MG rule for words ending with *"-ed" *(verb or adjective)
CD	number	NUMBERS	MG rule for numbers
RB	adverb, base	words.adv.1	ordinary manner adverbs

**Table 3:** Ambiguity for single extensions

Metric	Orig	UMLS	xMG	Brill	GT
Time	15.4s	9.9s	10.8s	8.8s	8.6s
Lkg. ratio	1	0.67	0.68	0.70	0.66

Time is average parsing time per sentence, linkage ratio is average of per-sentence linkage number ratios.

**Table 4:** Performance for single extensions

	Orig	UMLS	Δ	xMG	Δ	Brill	Δ	GT	Δ
All, first linkage	74.2	75.4	4.7	76.0	7.0	75.4	4.7	76.8	10.1
All, best linkage	82.7	83.5	4.6	84.5	10.4	83.7	5.8	85.3	15.0
NT, first linkage	78.0	78.1	0.5	78.9	4.1	78.0	0.0	79.4	6.4
NT, best linkage	87.4	86.9	-4.0	88.0	4.8	86.7	-5.6	88.3	7.1
*p*	N/A		*p *≈ 0.06		*p *< 0.01		*p *≈ 0.07		*p *< 0.01

First linkage denotes the linkage ordered first by the parser heuristics and best linkage the best performance achieved by any linkage returned by the parser. Results marked NT are for the subset of sentences where no timeouts occurred for any of the modifications. Δ columns give relative decrease in error with respect to the original LGP, and *p *values are for "All, first linkage" performance.

**Table 5:** Ambiguity for combinations of the extensions

Metric	Orig	UMLS & xMG	xMG & POS	UMLS & POS	All 3
Time	15.4s	9.5s	8.7s	8.3s	8.4s
Lkg. ratio	1	0.67	0.59	0.62	0.66

**Table 6:** Performance for combinations of the extensions

	Orig	UMLS & xMG	Δ	xMG & POS	Δ	UMLS & POS	Δ	All 3	Δ
All, first linkage	74.2	75.7	5.8	76.8	10.1	76.0	7.0	76.1	7.4
All, best linkage	82.7	83.7	5.8	85.3	15.0	84.2	8.7	84.2	8.7
NT, first linkage	78.0	78.4	1.8	79.3	5.9	78.6	2.7	78.7	3.2
NT, best linkage	87.4	87.0	-3.2	88.2	6.3	87.2	-1.6	87.1	-2.4
*p*	N/A		*p *< 0.05		*p *< 0.01		*p *< 0.01		*p *< 0.01

## References

[B1] Sekine S (1997). The Domain Dependence of Parsing. Proceedings of the 5th ACL Conference on Applied Natural Language Processing (ANLP'97).

[B2] Grishman R, Nebel B (2001). Adaptive Information Extraction and Sublanguage Analysis. Proceedings of the Workshop on Adaptive Text Extraction and Mining at the 17th International Joint Conference on Artificial Intelligence (IJCAI'01).

[B3] Lease M, Charniak E, Dale R, Wong KF, Su J, Kwong OY (2005). Parsing Biomedical Literature. Proceedings of the 2nd International Joint Conference on Natural Language Processing (IJCNLP'05).

[B4] Pyysalo S, Ginter F, Pahikkala T, Boberg J, Järvinen J, Salakoski T (2006). Evaluation of Two Dependency Parsers on Biomedical Corpus Targeted at Protein-Protein Interactions. Int J Med Inform.

[B5] Blaschke C, Andrade MA, Ouzounis CA, Valencia A, Lengauer T, Schneider R, Bork P, Brutlag DL, Glasgow JI, Mewes HW, Zimmer R (1999). Automatic Extraction of Biological Information from Scientific Text: Protein-Protein Interactions. Proceedings of the 7th International Conference on Intelligent Systems for Molecular Biology (ISMB'99).

[B6] Pustejovsky J, Castaño J, Zhang J, Kotecki M, Cochran B, Altman RB, Dunker AK, Hunter L, Lauderdale K, Klein TE (2002). Robust Relational Parsing over Biomedical Literature: Extracting Inhibit Relations. Proceedings of the 7th Pacific Symposium on Biocomputing (PSB'02).

[B7] Yakushiji A, Tateisi Y, Miyao Y, Tsujii J (2001). Event Extraction from Biomedical Papers Using a Full Parser. Proceedings of the 6th Pacific Symposium on Biocomputing (PSB'01).

[B8] Park J, Kim H, Kim J (2001). Bidirectional Incremental Parsing for Automatic Pathway Identication with Combinatory Categorial Grammar. Proceedings of the 6th Pacific Symposium on Biocomputing (PSB'01).

[B9] Clegg A, Shepherd A (2005). Evaluating and Integrating Treebank Parsers on a Biomedical Corpus. Proceedings of the Association for Computational Linguistics Workshop on Software.

[B10] Sleator DD, Temperley D (1991). Parsing English with a Link Grammar. Tech Rep CMU-CS-91-196.

[B11] Link Grammar. http://www.link.cs.cmu.edu/link/.

[B12] Ding J, Berleant D, Xu J, Fulmer AW, Werner B (2003). Extracting Biochemical Interactions from MEDLINE Using a Link Grammar Parser. Proceedings of the 15th IEEE International Conference on Tools with Artificial Intelligence.

[B13] Szolovits P, Musen M (2003). Adding a Medical Lexicon to an English Parser. Proceedings of the 2003 AMIA Annual Symposium.

[B14] Ahmed ST, Chidambaram D, Davulcu H, Baral C (2005). IntEx: A Syntactic Role Driven Protein-Protein Interaction Extractor for Bio-Medical Text. Proceedings of the ACL-ISMB Workshop on Linking Biological Literature, Ontologies and Databases: Mining Biological Semantics.

[B15] Alphonse E, Aubin S, Bessières P, Bisson G, Hamon T, Laguarigue S, Nazarenko A, Manine AP, Nédellec C, Vetah MOA, Poibeau T, Weissenbacher D, Collier N, Ruch P, Nazarenko A (2004). Event-Based Information Extraction for the Biomedical Domain: the Caderige Project. Proceedings of the COLING NLPBA/BioNLP Workshop.

[B16] The SPECIALIST NLP Tools. http://specialist.nlm.nih.gov/.

[B17] Aubin S, Nazarenko A, Nédellec C, Angelova G, Bontcheva K, Mitkov R, Nicolov N, Nikolov N (2005). Adapting a General Parser to a Sublanguage. Proceedings of the International Conference on Recent Advances in Natural Language Processing (RANLP'05).

[B18] Extending the Link Grammar Parser's lexicon from UMLS' Specialist lexicon. http://groups.csail.mit.edu/medg/projects/text/lexicon.html.

[B19] Mikheev A (1997). Automatic Rule Induction for Unknown-word Guessing. Computational Linguistics.

[B20] Spyns P (1994). A Robust Category Guesser for Dutch Medical Language. Proceedings of the 4th ACL Conference on Applied Natural Language Processing (ANLP'94).

[B21] Grover C, Lapata M, Lascarides A (2005). A Comparison of Parsing Technologies for the Biomedical Domain. Journal of Natural Language Engineering.

[B22] Brill Tagger. http://research.microsoft.com/users/brill/.

[B23] GENIA Tagger. http://www-tsujii.is.s.u-tokyo.ac.jp/GENIA/tagger/.

[B24] Tsuruoka Y, Tateishi Y, Kim JD, Ohta T, McNaught J, Ananiadou S, Tsujii J, Bozanis P, Houstis EN (2005). Developing a Robust Part-of-Speech Tagger for Biomedical Text. Proceedings of the 10th Panhellenic Conference on Informatics.

[B25] Kim JD, Ohta T, Tateisi Y, Tsujii J (2003). GENIA Corpus-a semantically annotated corpus for bio-textmining. Bioinformatics.

[B26] Kulick S, Bies A, Liberman M, Mandel M, McDonald R, Palmer M, Schein A, Ungar L (2004). Integrated Annotation for Biomedical Information Extraction. Proceedings of HLT/NAACL 2004 BioLink Workshop.

[B27] Database of Interacting Proteins. http://dip.doe-mbi.ucla.edu/.

[B28] Tsivtsivadze E, Pahikkala T, Pyysalo S, Boberg J, Mylläri A, Salakoski T, Famili AF, Kok JN, Peña JM, Siebes A, Feelders AJ (2005). Regularized Least-Squares for Parse Ranking. Proceedings of the 6th International Symposium on Intelligent Data Analysis (IDA'05).

[B29] Tsivtsivadze E, Pahikkala T, Boberg J, Salakoski T, Ali M, Dapoigny R (2006). Locality-Convolution Kernel and Its Application to Dependency Parse Ranking. Proceedings of the The 19th International Conference on Industrial, Engineering & Other Applications of Applied Intelligent Systems.

[B30] Wilcoxon F (1945). Individual Comparisons by Ranking Methods. Biometrics.

[B31] Demšar J (2006). Statistical Comparisons of Classifiers over Multiple Data Sets. Journal of Machine Learning Research.

[B32] BioLG. http://www.it.utu.fi/biolg.

